# Biomineralization Induced by *Colletotrichum acutatum*: A Potential Strategy for Cultural Relic Bioprotection

**DOI:** 10.3389/fmicb.2018.01884

**Published:** 2018-08-14

**Authors:** Tianxiao Li, Yulan Hu, Bingjian Zhang

**Affiliations:** Department of Cultural Heritage and Museology, Zhejiang University, Hangzhou, China

**Keywords:** biomineralization, *Colletotrichum acutatum*-induced calcium carbonate precipitation, mechanism, bioprotection, stone relics

## Abstract

*Colletotrichum acutatum* is a fungus capable of biomineralization reported in our previous study. In this paper, we compared the ability of this fungus to induce mineralization under different calcium sources, pH levels, and differing carbon availability. Here we found that organic acids, the alkalinity of the environment, and low carbon conditions were major factors influencing calcium carbonate precipitation. High performance liquid chromatography showed that citric acid was a metabolite produced by *C. acutatum*, and that other organic acids including formic, propionic, α-ketoglutaric, lactic, and succinic acids can be used by this fungus to promote CaCO_3_ formation. Based on these findings, the mechanism of the biomineralization induced by *C. acutatum* should be divided into three processes: secreting organic acid to dissolve limestone, utilizing the acid to increase the alkalinity of the microenvironment, and chelating free calcium ions with extracellular polymeric substances or the cell surface to provide a nucleation site. Interestingly, we found that hydroxyapatite rather than calcium carbonate could be produced by this fungus in the presence of phosphate. We also found that the presence of acetic acid could inhibit the transformation of vaterite to calcite. Further, we evaluated whether the proliferation of *C. acutatum* could influence the deterioration of stone relics. We found that low carbon conditions protected calcium carbonate from dissolution, indicating that the risk of degradation of limestone substrates caused by *C. acutatum* could be controlled if the fungi were used to consolidate or restore stone monuments. These results suggest that *C. acutatum-*induced biomineralization may be a useful treatment for deteriorated stone relics.

## Introduction

Outdoor stoneworks important to our shared cultural heritage are subject to physical, chemical, and biological weathering. Some minerals, such as calcium oxalates and calcium carbonates produced by microbial communities, have been shown to promote the protection of building stone from weathering (Monte et al., 1987; Salvadori and Municchia, 2016). Numerous microbial strains isolated from stone monuments can induce the precipitation of minerals there, and this type of biomineralization is a highly promising technique with the potential to remediate and protect stone relics (Muynck et al., 2010; Rodriguez-Navarro et al., 2011; Dhami et al., 2014; Gadd and Dyer, 2017).

Calcium carbonate and calcium oxalate are the most common minerals produced by microorganisms found on stone cultural monuments. The formation of calcium oxalate is mainly induced by oxalate acids secreted by microorganisms (Gadd et al., 2014). Most previous studies have reported that the class of microbes responsible for inducing the precipitation of calcium carbonate were heterotrophic bacteria, and these could be divided into three types according to their mechanism of biomineralization. These types include: sulfate reducing bacteria, bacteria utilizing organic acids, and bacteria involved in the nitrogen cycle (those that participate in the ammonification of amino acids, nitrate reduction, or hydrolysis of urea) (Dhami et al., 2013, 2014). In addition, fungi can also be responsible for CaCO_3_ production; in a previous study we found that *Colletotrichum acutatum*, a fungal pathogen of fruit crops, was capable of CaCO_3_ formation (Li et al., 2018). Similarly, other fungi, including *Fusarium oxysporum*, *Serpula himantioides*, and *Cephalotrichum*, have been found to take part in CaCO_3_ production (Burford et al., 2003; Rautaray et al., 2003; Li et al., 2015). Despite these findings, whether the fungus itself is responsible for the formation of calcium carbonate, the mechanism by which this may occur, and the factors involved in the process are not clear.

In recent years, bacterial-induced biomineralization has emerged as an environmentally friendly method for the effective protection and consolidation of decayed ornamental stoneworks (Muynck et al., 2010; Dhami et al., 2013, 2014). Using this method, precipitated calcium carbonates can cement loose rocks, restore weathered structures, and reduce water permeation and corrosion (Métayer-Levrel et al., 1999; Tittelboom et al., 2010; Phillips et al., 2013). However, few studies focus on the safety of bacterial-induced biomineralization for relic substrates. The growth and activity of living organisms, including the process of calcium carbonate precipitation, may lead to deterioration and discoloration of the relic (Scheerer et al., 2009). For example, ammonia produced by the hydrolysis of urea poses an environmental threat and causes staining (Tobler et al., 2011). Furthermore, acid secreted by organisms may solubilize carbonate minerals that are commonly used in many types of artwork and monuments (Sand, 1996). Thus, a thorough understanding of the risks involved in microbial biodeterioration is essential.

In our previous study, *C. acutatum* was isolated from the patina on Feilaifeng limestone and was identified as a fungus capable of inducing calcium carbonate precipitation. Here, we examined the metabolic activity of this fungus during its proliferation to evaluate whether it can be used *in situ* to protect the cultural relics. Next, we manipulated factors affecting incubation, such as the calcium source, pH and/or the types of organic acids available, to determine which factors affected biomineralization. Finally, CaCO_3_ and the fungi were cultured separately to simulate biodeterioration. These results help to clarify the mechanism of biomineralization of *C. acutatum* as well as factors that influence crystallization. These findings will help us to prevent from the occur of patina on the surface of Feilaifeng limestone and find an optimal bio-treatments for the protection and consolidation of cultural heritage.

## Materials and Methods

### Cultivation of Calcifying Fungi Under Various Conditions

Two media that provide organic and inorganic nutrients, respectively, were used to culture the calcifying fungi *C. acutatum*. These included B4 precipitation medium, which was composed of 2.5 g calcium acetate, 4 g yeast extract, and 10 g glucose per liter of water, and Czapek Dox Liquid Medium, which was composed of 2.5 g calcium acetate, 3 g NaNO_3_, 0.5 g KCl, 1 g K_2_HPO_4_, 0.5 g MgSO_4_⋅7H_2_O, 0.01 g FeSO_4_, and 20 g sucrose per liter watern. We made 21 kinds of media for further study by modifying the composition of these first two media. These modifications included changing the calcium source, adding urea, changing the pH, and/or removing the sugar (see **Supplementary Table S1** in the Supplement for a detailed list of media types used).

We cultured fungi using static cultivation in a 90 mm Petri dish for the group of 21 media, since our major limitation in this experimental design was space. For Group b samples (**Supplementary Table S1**), we analyzed the metabolic activity of the fungi by culturing in a 100 ml beaker flask shaken at 150 r/min. In addition, we designed a special culturing method (**Supplementary Figure S1**) for a medium that contained CaCO_3_, CaSO_4_, or calcium oxalate, which is hardly soluble in water: 1 g calcium salt powder was wrapped by two layers of cellophane to prevent the direct contact with fungi, then the complex was cultured with the media (**Supplementary Table S1**) full of fungi in a 100 ml beaker flask shaken at 150 r/min for 7 days.

*Colletotrichum acutatum* was the calcifying fungi evaluated in all samples in this study. Samples of this species were resuscitated from −20°C. The initial concentration of the fungi was adjusted to 1 × 10^5^CFU/ml prior to inoculation and fungal samples were incubated at 28°C for 7 days.

### Measurements of pH, Ca, and Organic Acids

For Group b samples, 5 ml samples of medium were extracted every 2 days and filtered through 0.22 um membranes. The sterilized medium was then stored at 4°C. Changes in the pH and in the calcium concentration of the medium were detected using an S220 Seven Compact pH/ion meter (Mettler Toledo, Switzerland). We found that there was a sharp increase in pH (except in Group b3) between the first and third days. Because this may reflect the utilization of organic acids, we used high performance liquid chromatography (HPLC) to determine the organic acids present in samples (**Supplementary Figure S2**). Considering the disturbance of acetas in Group b1 and b2 and the expense, only Group b4 medium taken on the first and third days were analyzed in the study. Five organic acids (acetic, lactic, citric, succinic, and α-ketoglutaric acids) were used as the control sample and 100 mg/L of them were also prepared for HPLC, which was performed using a WATERS 2690 Separation Module (GenTech, United States). The column temperature was 30°C and the mobile phase consisted of basic ammonium and methanol at a ratio of 99:1. The pH was adjusted to 2.6 with phosphoric acid at a flow rate of 0.5 ml/min.

### Biodeterioration Potential of CaCO_3_ by *C. acutatum*

As shown in **Supplementary Figure S1**, *C. acutatum* was cultured with CaCO_3_ for 7 days, where cellophane was used as a barrier. The components of the media are given in **Supplementary Table S1** and the media consisting of yeast extract and glucose were used as a control. After sterilization, a cellophane bag containing CaCO_3_ was dialyzed with deionized water for 24 h then dried at 60°C for 72 h. The weight of the bag was measured before and after the experiment, and the result was analyzed using R version 3.0.3^1^ Two-tailed *T*-tests were performed and *p* values <0.05 were considered to be statistically significant.

### Micrographical and Mineralogical Analyses of Crystals

**FIGURE 1:**
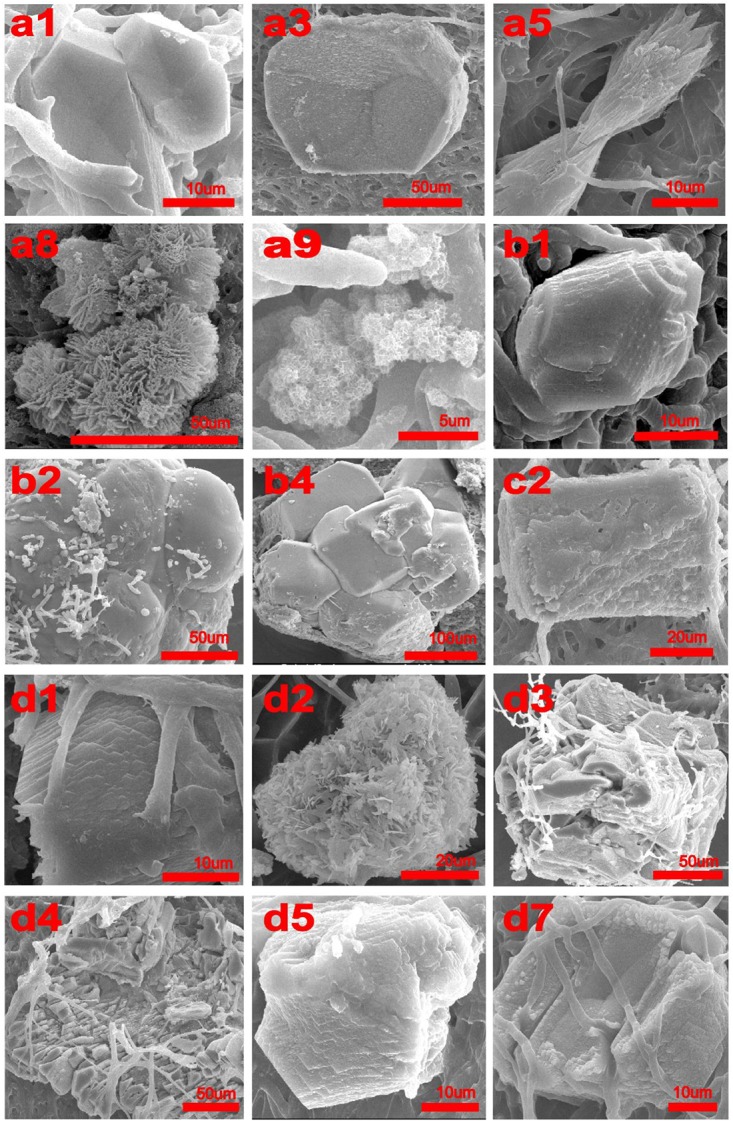
SEM micrographs of crystals produced by *Colletotrichum acutatum* with different culturing condition. **(a1)** calcium acetate in B4 medium, **(a3)** CaCl_2_ with urea in B4 medium, **(a5)** Ca(OH)_2_ in B4 medium, **(a8)** CaCl_2_ in Czapek-Dox medium, **(a9)** calcium acetate in Czapek-Dox medium, **(b1)** calcium acetate in B4 medium with shaking, **(b2)** calcium acetate in B4 medium without glucose, **(b4)** CaCl_2_ in B4 medium without glucose, **(c2)** CaCl_2_ in B4 medium with pH = 8, **(d1)** formic acid in B4 medium, **(d2)** propionic acid in B4 medium, **(d3)** α-Ketoglutaric acid in B4 medium, **(d4)** calcium lactate in B4 medium, **(d5)** cadmium succinate in B4 medium, **(d7)** calcium citrate in B4 medium.

An optical microscope was used to check whether crystals could be produced by *C. acutatum* in different conditions. The minerals produced were then analyzed using a scanning electron microscope (SEM, SIRION-100, Netherlands). Samples were cut into 2 mm × 2 mm pieces and examined by SEM equipped with an Energy-Dispersive Spectroscopy (EDS) probe (**Figure 1** and **Supplementary Figure S3**).

Minerals precipitated by selected fungi were further examined using an X-ray polycrystal diffractometer (Rigaku D/Max 2550, Japan). Biomasses containing crystals were ground and firmly compacted on the reverse side of an aluminum specimen holder held against a glass side. Then the samples were analyzed over the range 5–80° 2𝜃 at a scan rate of 1°/min in 0.1° increments.

## Results

### Factors Related to *C. acutatum* Biomineralization

Calcium acetate, CaCl_2_, CaSO_4_, Ca(OH)_2_, and CaCO_3_ were used as different calcium sources to evaluate the biomineralization ability of *C. acutatum*. We found that CaCO_3_ could crystallize with calcium acetate (**Figures 1a1,b1**, **2a1,b1**) or Ca(OH)_2_ (**Figures 1a5**, **2a5**) which suggests that organic acid content and pH are related to biomineralization. In addition, when urea was added to media in which CaCl_2_ was the calcium source, CaCO_3_ could also crystallize (**Figures 1a3**, **2a3**).

**FIGURE 2:**
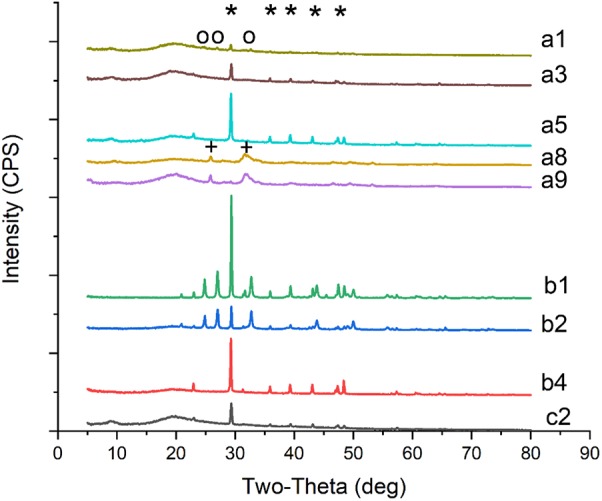
XRD patterns of crystals on biomass synthesized by *C. acutatum* in different conditions. (a1) calcium acetate in B4 medium, (a3) CaCl_2_ with urea in B4 medium, (a5) Ca(OH)_2_ in B4 medium, (a8) CaCl_2_ in Czapek-Dox medium, (a9) calcium acetate in Czapek-Dox medium, (b1) calcium acetate in B4 medium with shaking, (b2) calcium acetate in B4 medium without glucose, (b4) CaCl_2_ in B4 medium without glucose, (c2) CaCl_2_ in B4 medium with pH = 8; a strong amorphous peak observed at 2𝜃 around 20° may related to the glass background or the organic matter of fungi; ^∗^, the base peak of calcite; o, the peak of vaterite; +, the peak of hydroxyapatite.

**FIGURE 3:**
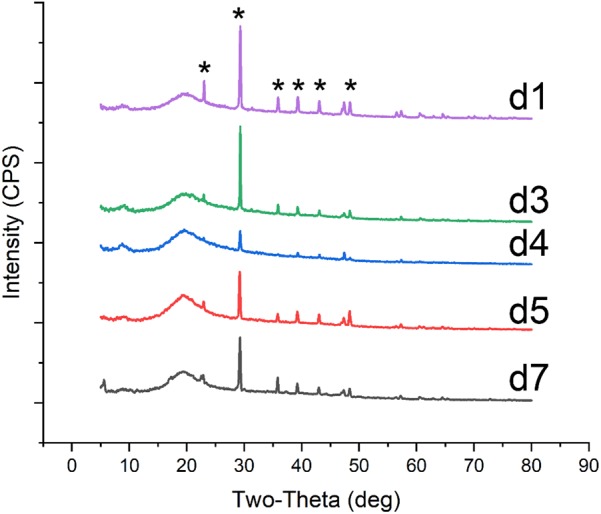
XRD patterns of crystals on biomass synthesized by *C. acutatum* with various organic acid. The mineral was produced in the condition (d1) formic acid, (d3)α-Ketoglutaric acid, (d4) calcium lactate, (d5) cadmium succinate, (d7) calcium citrate; a strong amorphous peak observed at 2𝜃 around 20° may related to the glass background or the organic matter of fungi; ^∗^, the base peak of calcite.

Interestingly, hydroxyapatite was observed among the fungi cultured with Czapek Dox medium (**Figures 1a8,a9**, **2a8,a9**), although the calcium source was CaCl_2_. The XRD results showed that hydroxyapatite was the major crystal formed by *C. acutatum* in Czapek Dox medium supplemented with calcium acetate (**Figure 2a9**), but CaCO_3_ was also found by SEM analysis. This showed that phosphate was better than organic acid in promoting biomineralization by *C. acutatum*.

**FIGURE 4:**
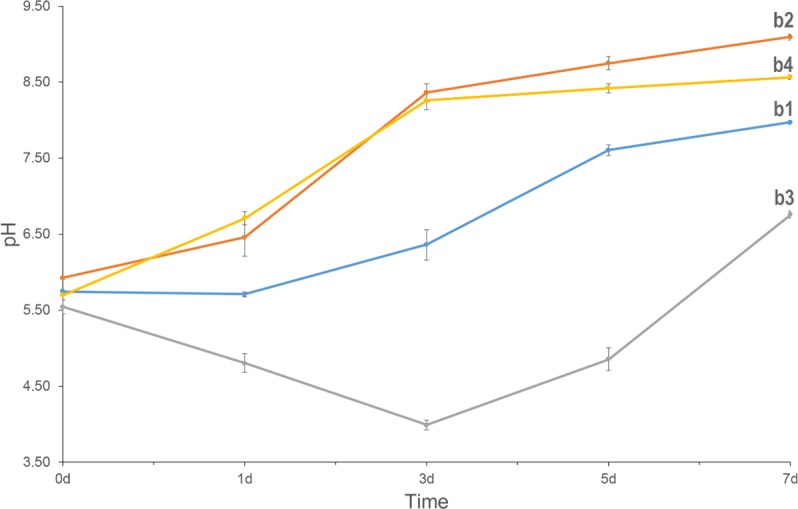
pH of the media with different conditions for the culturing of *C. acutatum*. (b1) Calcium acetate and glucose in the medium, (b2) calcium acetate in the medium, (b3) CaCl_2_ and glucose in the medium, (b4) CaCl_2_ in the medium.

After 7-day cultures, we observed pH rise in all media (**Figure 4**). Comparing Group b1 and b2, we found that low carbon source is better to promote pH increase. The pH of the CaCl_2_ control group dropped from 5.5 to 3.9 in the first three days but rose over the next 4 days. HPLC showed that there were four peaks in the medium from the first day and the retention times were 5.71, 7.02, 7.57, and 10.32, respectively. However, only three peaks were observed in the medium from the third day and the retention times were 5.17, 7.57, and 10.09. According to the HPLC results of five control acids, we could confirm that citric acid (6.96), α-ketoglutaric acids (7.83) were present in the first-day media, but α-ketoglutaric acids and lactic acid (10.07) were present in the third-day media. In addition, the amounts of α-ketoglutaric acids decreased. This indicated that the change in pH was likely related to the production and utilization of citric acid and α-ketoglutaric acids. In addition, CaCO_3_ was found in the biomass of all samples except those in the b3 Group (**Figures 1b1,b2,b4**, **2b1,b2,b4**). Group b2 was found to consume the greatest amount of calcium ions, followed by Group b4. These results suggest that *C. acutatum* can secrete organic acids, and that changes in pH may be related to the secretion and utilization of them. In addition, both organic acids and the low carbon condition may lead to CaCO_3_ formation, but the low carbon condition promote increased CaCO_3_ production.

When the fungus was cultured in media in which CaCl_2_ was used as the calcium source, CaCO_3_ was observed when the initial pH of the medium increased from 7 to 8 (**Figures 1c2**, **2c2**). Similarly, after adding urea to these media, we did not detect CaCO_3_ when the initial pH decreased to 7. This demonstrate that pH rather than the presence of urea promoted the formation of CaCO_3_.

### Biomineralization Induced by *C. acutatum* With Different Organic Acids

To examine differences in CaCO_3_ formation in the presence of different organic acids, acetic acid in the B4 medium was replaced by other seven organic acids or their salts. These included formic acid, propionic acid, α-ketoglutaric acid, calcium lactate, cadmium succinate, calcium oxalate, and calcium citrate, all of which are commonly produced by microbes. XRD and SEM results showed that all organic acids except oxalic acid were correlated with CaCO_3_ precipitation (**Figures 1d1–d5,d7**, **3**). Interestingly, the XRD result showed there is no CaCO_3_ in the biomass cultured with propionic acid, but a broken mineral containing Ca, C, and O was observed with SEM (**Figure 1d4** and **Supplementary Figure S3d4**). This may be amorphous calcium carbonate that cannot be identified by XRD. In addition, we found vaterite, an unstable crystal form of calcium carbonate, in the mineral precipitation formed in a culture containing acetic acid. These results demonstrate that vaterite and amorphous calcium carbonate can be stabilized by acetic and pyruvic acid, respectively.

### The Role of *C. acutatum* in CaCO_3_ Deterioration

**FIGURE 5:**
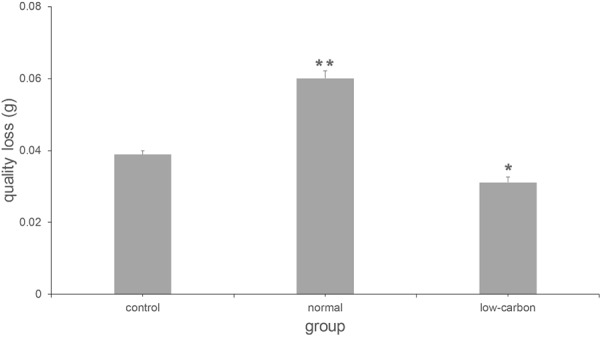
Quality loss of CaCO_3_ cultured with *C. acutatum* in different media. ^∗^*p* < 0.01; ^∗∗^*p* < 0.001.

**Figure 5** shows the mass-loss of CaCO_3_ cultured with *C. acutatum*. We found the weight of CaCO_3_ in the low carbon group is maximum, but that in the normal carbon group is minimum. This suggests that carbon deficiency, which is often present in the outdoor environment, may protect CaCO_3_ from damage.

## Discussion

In this study, a comprehensive examination according to the reported bacterial calcifying pathway was performed to estimate the mechanism of biomineralization induced by *C. acutatum*, a fungus first linked to calcium carbonate precipitation in our earlier investigation (Li et al., 2018). We found that the presence of phosphate, an alkaline environment, the presence of organic acids, and the lack of carbon can promote the formation of minerals among mycelia. In addition, the deterioration of CaCO_3_ can be inhibited by *C. acutatum* in low carbon conditions. These findings provide a new strategy for the prevention of biodeterioration and patina discoloration caused by *C. acutatum* on cultural relics. The mechanism of biomineralization induced by this fungus therefore presents a new method of bioprotection of stone relics.

Sufficient calcium and carbonate ions are required for CaCO_3_ formation. There are four key factors that govern the process of calcium carbonate precipitation: (1) calcium concentration, (2) the concentration of dissolved inorganic carbon (DIC), (3) pH, and (4) the availability of nucleation sites (Muynck et al., 2010; Dhami et al., 2014). For biomineralization to occur on the surface of limestone, calcium comes from the dissolution of carbonate by acid rain or by acids produced by colonizing microbial communities (Sand, 1996; Scheerer et al., 2009). In the present study, citric acid and α-ketoglutaric acid, the intermediate metabolites of the tricarboxylic acid cycle, were found in the medium cultured with *C. acutatum*. In addition, the loss of CaCO_3_ was observed in Group a6, which showed the possibility of CaCO_3_ decomposition and calcium release caused by the presence of fungi. However, in a low carbon condition similar to the outdoor environment, the dissolution of CaCO_3_ was inhibited. This result implies that *C. acutatum* cannot obtain enough calcium by itself for the precipitation of CaCO_3_ in nature. It is known that the amount of carbonate ions present is limited by the amount of DIC and pH in a given aquatic system (Dhami et al., 2013). Previous studies have demonstrated that during the biomineralization of CaCO_3_, the primary role of bacteria is to create an alkaline environment (Dhami et al., 2014). They do so by various metabolic activities, including sulfate reduction, organic acid degradation, and urea hydrolysis (Muynck et al., 2010; Dhami et al., 2014). In addition, carbon dioxide or bicarbonate is produced as a byproduct in this process which leads to an increase in the amount of DIC (Mitchell and Ferris, 2006). For *C. acutatum*, a similar mechanism was found regarding the precipitation of calcium carbonate. When acetate was added to media, CaCO_3_ could be observed within the mycelium and the incubated environment changed from acidic to alkaline. The production of CaCO_3_ and an accompanying increase in pH also occurred as glucose was removed. We found that in a low carbon condition, citric acid was produced by *C. acutatum* but was metabolized later followed by a pH increase. This finding shows that organic acids secreted by the fungi can act as carbon sources that it itself can further utilize, and that this raises the environmental pH.

We also tested whether urea and sulfate could be used for mineralization of calcium carbonate by fungi, but we found that no CaCO_3_ formed on the biomass if the initial media was acidic or had a neutral pH. These results show that an alkaline environment suitable for calcium carbonate precipitation can be produced by *C. acutatum* by using its own organic acids, and the lowest pH for effective biomineralization was 8. In addition, the consumption of these acids could result in the production of CO_2_ and OH^−^, which promotes the formation of HCO_3_^−^ (Knorre and Krumbein, 2000). According to the pH change of Group b3, there might be a lower utilization of organic acids when the carbon source was rich, then calcium carbonate could not precipitate under the low concentration of DIC. However, the carbon source would be sufficient to promote the activities of fungi, especially the respiration. Therefore, when the OH^−^ was added into the media, we could also observe the calcium carbonate precipitation (**Figure 1c2**). In synthesis, the presence of carbon dioxide along with the degradation of acids and respiration provided enough DIC for CaCO_3_ production. Extracellular polymeric substances (EPS) are metabolic products that accumulate on the microbial cell surface. These consist of many negatively charged functional groups including carbonyl, carboxyl, hydroxyl, and carbonyl groups (Ortegamorales et al., 2001). Such groups could serve as carbon and energy reserves during starvation and can also chelate free cations with their negative ions (Braissant et al., 2007). In addition, there are several negatively charged groups on the cell surface, and these can also trap calcium ions (Douglas and Beveridge, 1998; Bauerlein, 2010). During CaCO_3_ biomineralization, when the calcium is attracted, EPS or cell walls are believed to be the main nucleation sites for the formation of calcium carbonate (Rodriguez-Navarro et al., 2007; Riding, 2010). In the present study, citric acid and α-ketoglutaric acid were secreted outside the cell; they therefore may take part in the formation of EPS. The anions from the organic acids can promote the accumulation of calcium ions, and therefore EPS provide rich sites for CaCO_3_ precipitation. In summary, the production and consumption of organic acids were key to CaCO_3_ formation induced by *C. acutatum*.

Hydroxyapatite is a main constituent of hard tissues such as bone and teeth. There are several advantages to substrate consolidation when hydroxyapatite is applied to carbonate stones. These advantages include low viscosity, a lack of discoloration and/or changes in physical properties, and enhanced durability to variable temperature and salt crystallization (Sassoni et al., 2011). Given these properties, hydroxyapatite is widely used for the conservation of cultural relics (Sassoni, 2018). During the recent past years, many methods have been used to prepare hydroxyapatite, and many of these involve the reaction of calcium-rich substrates with an aqueous solution of a phosphate salt (Sadat-Shojai et al., 2013). In the present study, soluble calcium and phosphate were added to various media, which makes hydroxyapatite formation possible if the liquid environment is alkaline. *C. acutatum* can promote pH increases by using organic acids, and this may be the cause of the formation of hydroxyapatite in this study. This result therefore suggests another method for the preparation of hydroxyapatite. Comparing the formation of this mineral with the participation of CaCl_2_, we found that hydroxyapatite crystallizes easier than calcium carbonate in *C. acutatum*-induced biomineralization.

In our previous study, we reported that *C. acutatum* could suppress the transformation of vaterite to calcite (Li et al., 2018). This result was confirmed by the present study. Interestingly, vaterite was only observed in mineralization systems in which calcium acetate acted as the carbon source. Wada et al. (2001) evaluated the effect of seven carboxylic acids on the crystallization of calcium carbonate, but they did not assess the transformation between calcite and vaterite. In addition, amino acids, proteins, or sugars metabolized by microorganisms were validated by their ability to inhibit the transformation of vaterite to calcite (Wang et al., 2006). In this study, acetate can be used by *C. acutatum* for proliferation, which indicates that the metabolites during utilization of acetate may play a role in the control of the transformation between calcite and vaterite. Taken together, this suggests that acetate is related to the transformation of the crystal type of calcium carbonate, but a more detailed understanding of the mechanism by which this takes place should be examined in future studies.

Recent studies have identified microbial-induced calcium carbonate and hydroxyapatite precipitation as a promising form of bioremediation for culturally significant stone monuments. However, there are significant limitations, such as the fact that the growth and metabolism of microorganisms can lead to physical or chemical damage to the stone, and the fact that low quantities of microorganisms may not reach the threshold of effective consolidation (Dhami et al., 2013, 2014). In addition, the introduction of microorganisms may break the local environmental balance or have a risk for human health. In this study, we examined the protection offered by *C. acutatum* for CaCO_3_ and confirmed the safety of the fungi used to consolidate limestone. In addition, the mechanism of biomineralization as well as important factors contributing to biomineralization caused by fungi were also systematically tested. The knowledge gained here may be helpful for the development of biological preservation treatments involving this fungus, but we should not ignore its safety for environment or human before application. Overall, we believe that *C. acutatum* may be useful for the protection of cultural relics in the future. In addition, the findings will be useful for the understanding the applications of bio-treatment in other areas, such as self-healing concrete (Luo et al., 2018) and bio-consolidation (DeJong, et al., 2010).

## Conclusion

We clarified the mechanism of calcium carbonate precipitation induced by *C. acutatum*, a fungus first reported as capable of biomineralization in a previous study performed by our lab. After producing and consuming organic acids such as citric acid, this fungus generates byproducts that increase the pH and the concentration of DIC. The EPS or cell surface, both of which are rich in free calcium ions, provide nucleation sites for CaCO_3_ formation. In addition, this process can also produce hydroxyapatite in the presence of phosphate. Initial pH levels higher than 8 is an essential prerequisite for calcium carbonate precipitation, and the presence of organic acid and low carbon conditions influence the amount of CaCO_3_ produced by fungal biomineralization. Vaterite can remain in a stable state with the influence of *C. acutatum* if enough acetic acid is present in the living environment. Finally, low carbon conditions can protect calcium carbonate by inhibiting its degradation. The controllable amounts of CaCO_3_ and the safety of the fungal biomineralization procedure illustrate that *C. acutatum*-induced biomineralization may be an ideal treatment for the preservation of deteriorated stone relics.

## Author Contributions

TL, YH, and BZ performed the experiments and analyzed the data. TL wrote the manuscript. YH and BZ participated to the revision of the manuscript.

## Conflict of Interest Statement

The authors declare that the research was conducted in the absence of any commercial or financial relationships that could be construed as a potential conflict of interest.

## Footnotes

1 www.r-project.org
