# Supplementary material for: Biomineralization Induced by Colletotrichum acutatum: A Potential Strategy for Cultural Relic Bioprotection
Source: Front Microbiol. 2018 Aug 14;9:1884. doi: 10.3389/fmicb.2018.01884 (PMC6104437; doi:10.3389/fmicb.2018.01884)
Supplement: Supplementary file 1 [file Data_Sheet_1.docx]

*Supplement of*

**Biomineralization induced by *Colletotrichum acutatum*: a potential strategy for cultural relic bioprotection**

**Tianxiao Li et al.**

*Correspondence to:* Bingjian Zhang (zhangbiji@zju.edu.cn)

**Supplement Contents**

This supplement contains, in the following order:

Table S1. The group set of the various media with different condition.

Figure S1. The way of insoluble calcium source cultured with *Colletotrichum acutatum*.

Figure S2. HPLC of the media cultured with *Colletotrichum acutatum* in the low carbon condition with CaCl_2_ at the first and third day.

Figure S3. EDS analysis of the precipitated crystals.

Table S1: The group set of the various media with different condition.

| group | Ca2+ | B4 | | |  | Czapek-Dox Medium | Other additives | Initial pH |
| --- | --- | --- | --- | --- | --- | --- | --- | --- |
|  |  | yeast extract | | glucose |  |  |  |  |
| a1 | calcium acetate | + | + | |  | - | - | 5.7 |
| a2 | CaCl_2_ | + | + | |  | - | - | 5.5 |
| a3 | CaCl_2_ | + | + | |  | - | Urea | 8.0 |
| a4^*^ | CaSO_4_ | + | + | |  | - | - | 5.5 |
| a5 | Ca(OH)_2_ | + | + | |  | - | - | 12.0 |
| a6^*^ | CaCO_3_ | + | + | |  | - | - | 5.5 |
| a7^*^ | CaCO_3_ | + | | - |  | - | - | 5.5 |
| a8 | CaCl_2_ | - | - | |  | + | - | 5.4 |
| a9 | calcium acetate | - | - | |  | + | - | 5.4 |
| b1^*^ | calcium acetate | + | + | |  | - | - | 5.7 |
| b2^*^ | calcium acetate | + | | - |  | - | - | 5.9 |
| b3^*^ | CaCl_2_ | + | + | |  | - | - | 5.5 |
| b4^*^ | CaCl_2_ | + | | - |  | - | - | 5.5 |
| c1 | CaCl_2_ | + | + | |  | - | NaOH | 7.0 |
| c2 | CaCl_2_ | + | + | |  | - | NaOH | 8.0 |
| c3 | CaCl_2_ | + | + | |  | - | Urea, HCl | 7.0 |
| d1 | formic acid | + | + | |  | - | Ca(OH)_2_ | 7.0 |
| d2 | propionic acid | + | + | |  | - | Ca(OH)_2_ | 7.0 |
| d3 | α-ketoglutaric acid | + | + | |  | - | Ca(OH)_2_ | 7.0 |
| d4 | calcium lactate | + | + | |  | - | - | 7.0 |
| d5 | cadmium succinate | + | + | |  | - | - | 7.0 |
| d6^*^ | calcium oxalate | + | + | |  | - | - | 7.0 |
| d7 | calcium citrate | + | + | |  | - | - | 7.0 |

+: the component was added into the media; -: the substance was removed from the media; *: the media were cultured with shaking


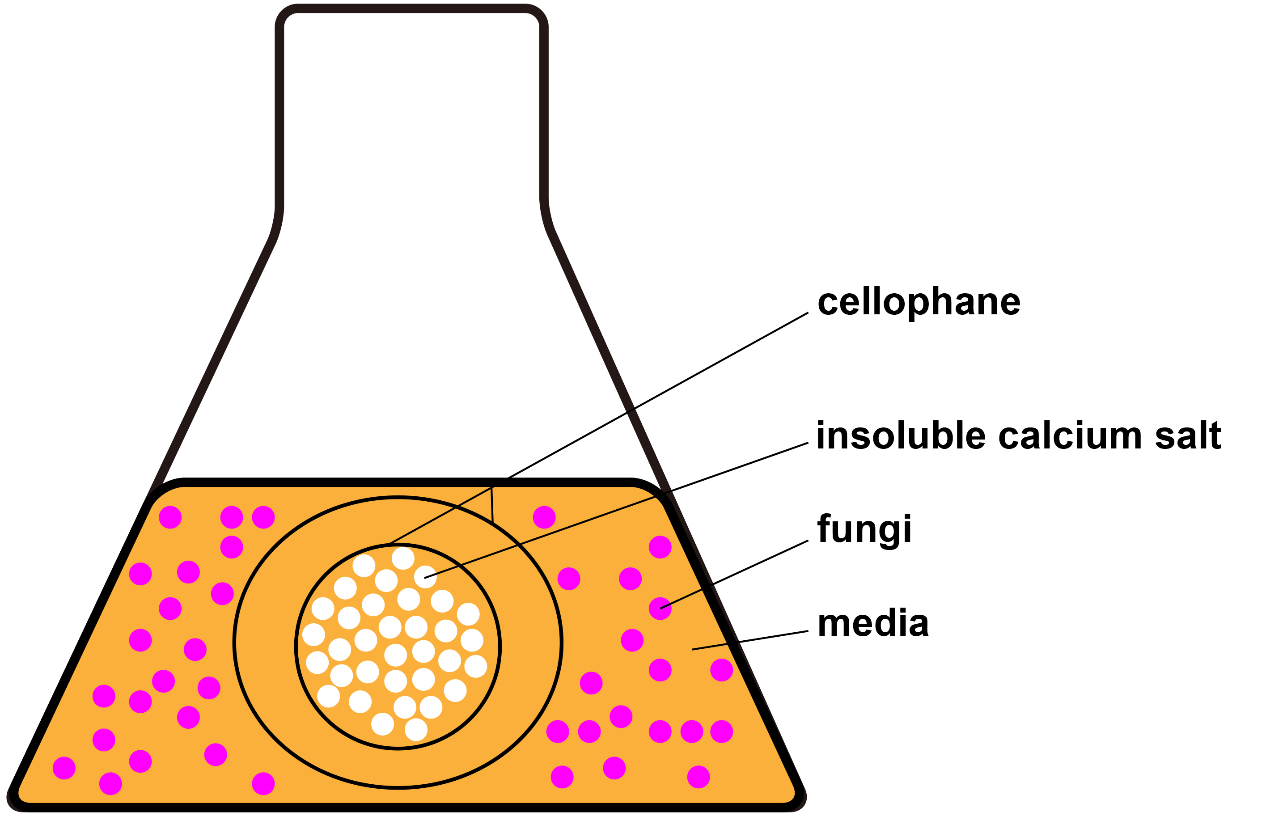


Figure S1: The way of insoluble calcium source cultured with *Colletotrichum acutatum*.


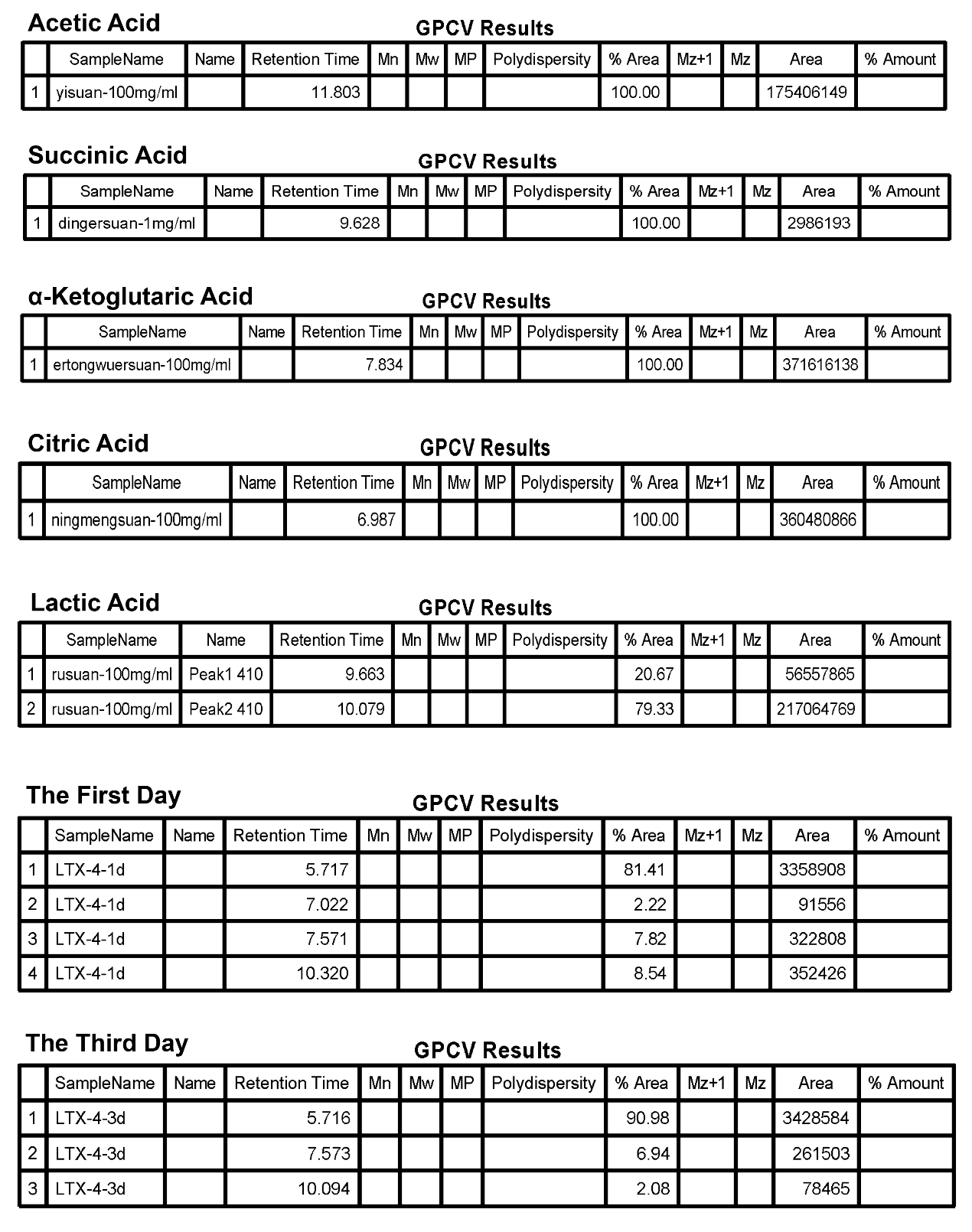


Figure S2: HPLC of the media cultured with *Colletotrichum acutatum* in the low carbon condition with CaCl_2_ at the first and third day.


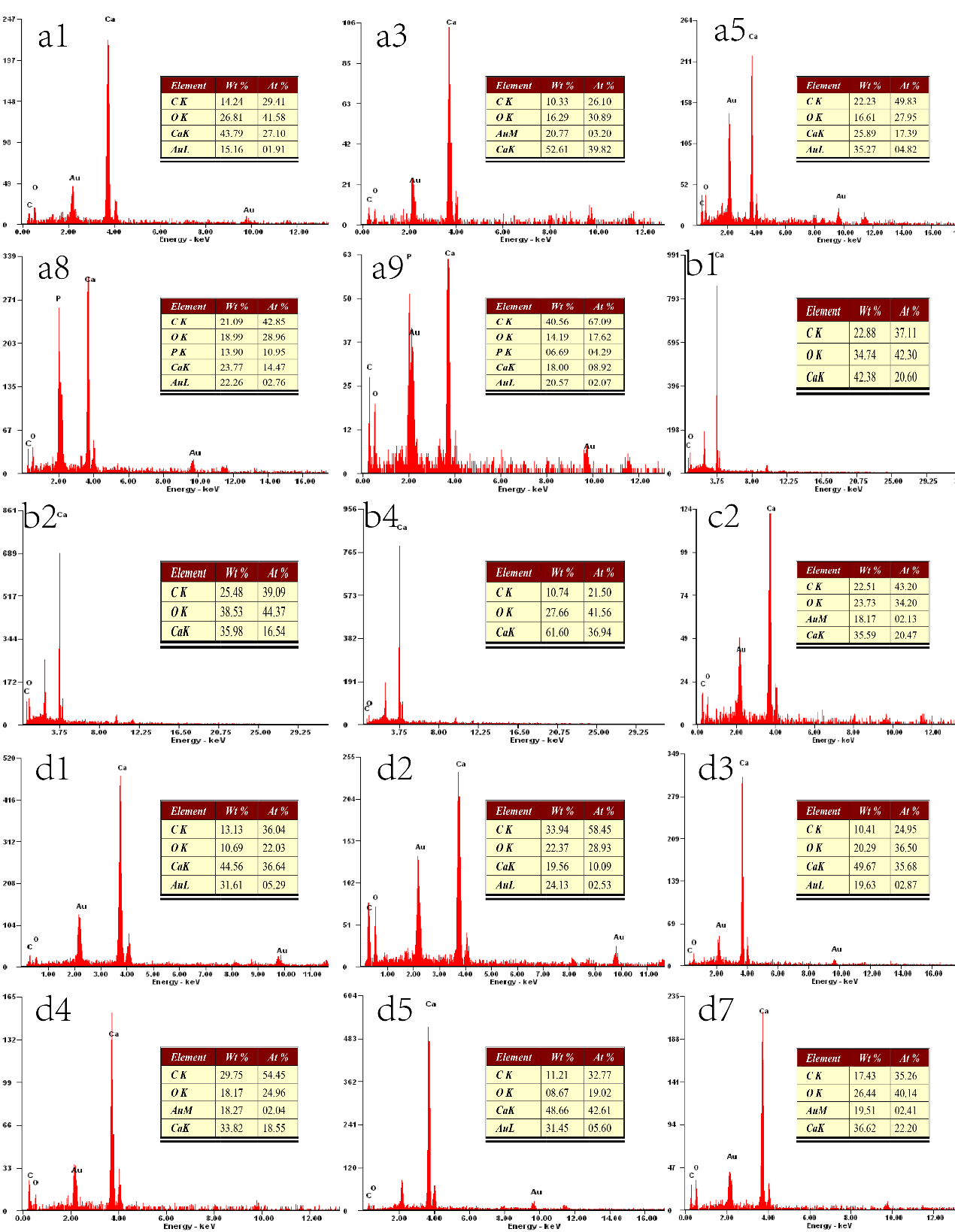


Figure S3: EDS analysis of the precipitated crystals. (a1) calcium acetate in B4 medium, (a3) CaCl_2_ with urea in B4 medium, (a5) Ca(OH)_2_ in B4 medium, (a8) CaCl_2_ in Czapek-Dox medium, (a9) calcium acetate in Czapek-Dox medium, (b1) calcium acetate in B4 medium with shaking, (b2) calcium acetate in B4 medium without glucose, (b4) CaCl_2_ in B4 medium without glucose, (c2) CaCl_2_ in B4 medium with pH=8, (d1) formic acid in B4 medium, (d2) propionic acid in B4 medium, (d3) α-Ketoglutaric acid in B4 medium, (d4) calcium lactate in B4 medium, (d5) cadmium succinate in B4 medium, (d7) calcium citrate in B4 medium.
